# The Impact of COVID-19 Pandemic on the Castile and Leon Addiction Treatment Network: A Real-Word Experience

**DOI:** 10.3389/fpsyt.2020.575755

**Published:** 2020-11-25

**Authors:** Carlos Roncero, Begoña Vicente-Hernández, Nerea M. Casado-Espada, Lourdes Aguilar, Sinta Gamonal-Limcaoco, María A. Garzón, Fernando Martínez-González, Carlos Llanes-Álvarez, Ruth Martínez, Manuel Franco-Martín, Ana Álvarez-Navares

**Affiliations:** ^1^Psychiatry Service, University of Salamanca Health Care Complex, Salamanca, Spain; ^2^Institute of Biomedicine of Salamanca (IBSAL), University of Salamanca, Salamanca, Spain; ^3^Psychiatry Unit, School of Medicine, University of Salamanca, Salamanca, Spain; ^4^Addictions and Dual Disorders Unit, Psychiatry Service, Salamanca University Health Care Complex, Salamanca, Spain; ^5^Regional Commissioner for Drugs, Social Services Management, Castile and Leon Regional Government, Valladolid, Spain; ^6^Psychiatry Service, Zamora Health Care Complex, Zamora, Spain; ^7^Psychiatry Service, Rio Hortega University Hospital, Valladolid, Spain

**Keywords:** COVID-19, impact, network on drugs and drug addiction, assistance, relapse

## Abstract

**Background:** Patients suffering from addiction are a vulnerable group in the midst of COVID-19, so their healthcare is considered essential. In this paper, the measures and responses of the Drug Addiction Assistance Network of Castile and Leon (DAACYL) in Spain during the first 6 weeks of the COVID-19 pandemic are explained. The aim is that this experience could be useful in places where this problem will continue and could help future interventions.

**Methods:** A telephone survey was carried out as the main methodology, to collect information for the subsequent organization and repercussion on professionals and patients. This was carried out by the heads of the 18 DAACYL units. Among the interventions applied, the following stand out: implantation of telemedicine techniques, restriction of daily methadone dispensing, suspension of urine controls and initiation of care programs for the homeless.

**Results:** As a result of these interventions, the professionals observed that patients are less demanding and mostly stable, with a low percentage of relapses. An increase in the consumption of alcohol and benzodiazepines have been reported as more common among people who relapse. Furthermore, the prevalence of COVID-19 infection in the sample is minimal; therefore, different hypotheses should be considered as an explanation (infra-diagnosis, immune system used to aggression, possible anti-inflammatory effect of some psychotropic drugs and a greater perception of danger against infection than the general population).

**Conclusions:** The rapid adaptation and successful implementation of DAACYL have had satisfactory results. On the other hand, the prevention of the possible increase in the development of behavioral addictions and the use of homemade drugs should be considered.

## Introduction

Since December 2019, Wuhan, China, reported cases of an acute respiratory disease. The cause was identified as a new coronavirus, previously unknown in humans, named COVID-19, which produces a syndrome called Severe Acute Respiratory Syndrome coronavirus 2 (SARS-CoV-2) (1). The characteristics of this disease include, aside from the pulmonary manifestations, the affectation of other organs (2), including the CNS (3). One of the main aspects of this virus is that it is very easily transmitted between people (2). Therefore, from China the infection has spread very quickly to other countries in Asia, Europe, Australia, Africa, and the American continent. During the writing of this article, the disease was declared in 207,634 people in Spain, of whom 23,190 died (4). Therefore, once the pandemic was declared in Spain, the work of reorganization of health and socio-health services, including mental health care was executed, just like China suggested (2, 5).

This infection can produce mental disorders in the general population (6, 7) and in the psychiatric community (6–9), including patients with addictions (10). This last patients, in addition to the risks related to mental health patients who, very frequently, smoke tobacco (9), many of them have respiratory problems due to the consumption of opiates, cannabis or other substances through the intrapulmonary route (10). Also, the presence of previous medical disorders is a risk factor associated with a higher risk of suicide and negative affects (6).

People with mental disorders have a higher risk of getting infected due to the lower ability to protect themselves, considering that in some cases they also show less self-control (5). In addition, specific problems should be considered to patients suffering from addiction must be considered, as they use intravenous drugs, and have partial access, or even barriers, to access treatment resources (11). Moreover, patients with a substance disorders have a greater risk of worsening their previous medical problems, including other infections (12). On the other hand, there is also a risk of overdosing when buying more adulterated or elaborated substances at home. The greater risk of infection rises due to stigmatization and social exclusion, sharing the material or being in risky environments (11, 13, 14).

Finally, it is considered that some consumers are homeless. This population has associated risks such as older age and the presence of medical diseases, as well as more difficulties accessing the health system, or carrying out preventive measures like social isolation, even if they have symptoms (15).

However, the treatment of the population with addiction is important because these patients can present disruptive behaviors, withdrawal syndromes that require healthcare, a risk of overdose, or even the inability to do a confinement due to their homeless situation (10, 16). There could even be a risk of developing behavioral addictions during the confinement, plus the difficulty to access illegal substances, could increase the possibility of creating homemade drugs which could have a greater toxicity (14).

The confinement situation declared by the Spanish government, in the Royal Decree 463/2020 of March 14, socio-sanitary assistance for people who use drugs has been considered a first necessity in Castile and León (Spain). People suffering from addiction have been recognized as a vulnerable group in the pandemic, their assistance being closely linked to COVID-19 (As dictated in the Instructions 1/2020 and 2/2020 of the Directorate of Legal Services of the Ministry of the Presidency of the Castile and Leon Regional Government. on suspension of deadlines of public sector procedures during the state of alarm) (17). This recognition has been corroborated and reinforced by the Resolution in April 8, 2020, of the Presidency of the Board of Directors of the Management of Social Services of Castile y León (18), by which specific procedural rules are determined, as a consequence of the declaration of the alarm status by COVID-19. This resolution establishes that patients are especially vulnerable to the effects of the pandemic and that healthcare services have to provide these people with basic social care (19). This is complemented by a contingency plan of the Regional Commissioner for drugs, released before the declaration of the state of alarm in order to adapt the healthcare response in Castile and Leon to the drug-dependent population to the restrictions and recommendations of the Castile and Leon Health authorities. In the international and national level, adjustments have been proposed for the drug addiction care programs (20–23).

The objective of this work is to describe the real word experience of the Castile and Leon Addiction Treatment Network (DAACYL), to the infection of COVID-19 and the repercussions detected in the first 6 weeks of the state of alarm.

## Materials and Methods

Castile and Leon is one of the Autonomous Communities of Spain, it has 2,393,285 inhabitants (24). The estimation is that more than 14,000 patients receive drug dependence treatments annually in the public health system, of which 5,300 have an alcohol addiction and more than 3,000 a nicotine addiction (25). The characteristics of this population are described in Table 1.

**Table 1:** The basic profile of patients with drug addiction treated at DAACYL 2019.

**Center or department**	***N***	**Sex %**		**Mean age (years)**	**Main drug %**					
		**Man**	**Woman**		**Heroin**	**Cocaine**	**Cannabis**	**Alcohol**	**Tobacco**	**Other**
FLSS-alcohol	2.493	78.8	21.2	Not available	0	0	0	100	0	0
FLSS for all drug	2.772	83.7	16.3	Not available	13.5	28.8	28.0	19.9	0	9.8
ODC	3.897	83.5	16.5	38.5	35.3	20.2	12.7	14.5	0	17.3
Day centers	1.026	75.9	24.1	45.0	6.4	19.6	14.2	53.1	0	6.7
OAC	1.246	83.8	16.2	49	0	8	0	92	0	0
OPDD	27	66.6	33.3	35.7	7.4	33.3	44.4	0	0	14.8
Group smoking dishabituation (AECC)	1.018	42.7	57.3	42.1	0	0	0	0	100	0
Smoking treatment units/consultations SACyL	2.332	48.7	51.3	Not available	0	0	0	0	100	0
ID-DDU	183	74.9	25.1	45.3	21.3	19.7	7.6	29.0	0	22.4
Therapeutic communities	565	87.5	12.5	38.9	12.3	46.0	7.8	14.4	0	19.5
ARC	232	86.9	13.1	46.6	0	0	0	100	0	0

*Drug Addiction Assistance Network of Castile and Leon (DAACYL). First level-specific services (FLSS). Outpatient treatment for patients with drug addiction (ODC). Outpatient alcohol clinic (OAC). Outpatient Program for Dual Disorder (OPDD). Tobacco treatment program (AECC). Inpatient detoxification and dual disorder unit (ID-DDU). Alcoholic Rehabilitation Centers (ARC)*.

The Drug Addiction Assistance of Castile and Leon (DAACYL) has around 400 professionals, including graduates in psychology, work and social education, medicine and nursing, who are the most numerous (25). This includes 27 first-level specific services (FLSS), of which 13 are exclusively for people with an alcohol use disorder, 11 outpatient drug clinics (ODC). Eight days care centers, one of them is specifically for alcoholics, two outpatient alcohol clinics (OAC), two outpatient dual disorder programs in Salamanca and Zamora integrated into the psychiatric services, 9 Spanish Network against Lung Cancer (AECC) tobacco treatment programs and 5 Tobacco Units/consultations. At the residential level, the specific network has a reference inpatient detoxification and dual disorder unit (ID-DDU) for the whole of Castile and León, located in Salamanca, with 24 professionals: 7 therapeutic communities (TC) and 2 alcoholic rehabilitation centers (ARC).

The centers are distributed throughout the area (Table 2), and their function and accessibility is described in the VII Regional Plan on Drugs 2017–2021 (26). In some cases these centers belong to the Psychiatric Department and are mostly managed by different non-profit entities in the third sector.

**Table 2:** Resources of the network for addiction treatment in castile and Leon (DDACYL) (Spain).

	**Ávila**	**Burgos**	**León**	**Palencia**	**Salamanca**	**Segovia**	**Soria**	**Valladolid**	**Zamora**	**Total**
FLSS for all drug patients with addiction	**1** Cáritas	**3** ACLAD Cáritas (A. de Duero) BOREAL (M. de Ebro)	**2** ACLAD Cáritas	**2** ACLAD ASCAT (Guardo)	**3** Cáritas apared nueva gente	**1** Cáritas		**2** ACLAD Cáritas		**14**
FLSS for alcoholics (associations for rehabilitated alcoholics)	**1** Geara	**2** ARBU AREMI (M. de Ebro)	**3** ARLE BEDA (Ponferrada) ARBA (La Bañeza)	**2** ARPA ARGU (Guardo)	**3** ARSA ARBE (Béjar) ARCIU (C. Rodrigo)	**1** ARSEG	**1** ARESO	**3** ARVA AVAR ATRA	**1** ARZA	**17**
ODC	**1** Cáritas	**1** Red cross	**2** Red cross Consejo comarcal de el bierzo (Ponferrada)	**1** S. JUAN DE DIOS	**1** Red cross	**1** Red cross	**1** Red cross	**2** Red cross ACLAD	**1** Cáritas	**11**
Day centers		**2** ARBU (alcohol dependents) PROYECTO HOMBRE	**2** PROYECTO HOMBRE of León y Ponferrada		**2** Cáritas proyecto hombre of Salamanca			**2** ACLAD proyecto hombre		**8**
Outpatient alcohol clinics (OAC)					**1** SACastilla y León				**1** SACastilla y León	**2**
Tobacco treatment programs	**1** AECC	**1** AECC	**1** AECC	**1** AECC	**1** AECC	**1** AECC	**1** AECC	**1** AECC	**1** AECC	**9**
Smoking units and cosultations		**1** SACastilla y León		**1** SACastilla y León	**1** SACASTILLA Y LEÓN			**1** SACastilla y León	**1** SACastilla y León	**5**
Inpatient detoxification and dual disorder units (ID-DDU)					**1** SACastilla y León					**1**
Therapeutic communities		**1** Proyecto hombre	**1** Proyecto hombre	**2** S. Juan de dios spiral	**1** Proyecto hombre (Salamanca)			**1** Proyecto hombre	**1** Cáritas	**7**
Alcoholic rehabilitation center (ARC)				**1** ALDAMA					**1** Cáritas	**2**
Total	4	11	11	10	14	4	3	12	7	76

*Roles of the different units*.

*First level specific services (FLSS): (1) information and guidance on the available resources, (2) recruitment, motivation, referral and psychosocial support for outpatient treatment, (3) coordination, support and development of the individualized social integration program. Specific centers for outpatient drug clinic (ODC): (1) outpatient treatment for drug dependent patients, (2) coordination, support and development of the individualized social integration program. Day centers: (1) treatment for patientsin an intermediate regime, (2) coordination, support and development of the individualized social integration program. Outpatient alcohol clinic (OAC): (1) outpatient treatment of alcoholism and mental disorders associated with alcohol dependence (referral service for Mental Health Teams in the Health Area). AECC tobacco treatment program: individual and group treatment (preferred) to quit smoking. Smoking units/consultations: individual treatment for smoking in a specialized level. Inpatient detoxification and dual disorder unit (ID-DDU): hospital detoxification for patients with addiction and hospital care for patients with dual disorders. Therapeutic communities: treatment of patients in a residential regime. Alcoholic Rehabilitation Centers (ARC): Treatment for alcoholics in a residential regime*.

A semi-structured telephone survey was carried out on April 13, 14, 15, 21, 22, and 24, by a RADCYL psychiatrist to each of 19 heads of the centers that make up the network, without exclusion criteria: 11 ODC of Castile and León, 2 OAC, 2 TC, the 2 outpatient programs of dual disorder and 2 ARC, following a structured guide of questions in which the following questions about the work system were addressed (Table 3): the impact of the pandemic on the organization of these centers and the repercussion on professionals and patients. The information on the other units (day centers, outpatient units, foster homes, etc.) was also collected.

**Table 3:** Phone interview guide.

• In the Outpatient Centers: ◦ Face-to-face assistance/telephone contact. ◦ Dispensing methadone and performing urine controls. ◦ Initiation of new treatments with opiate agonists (methadone, buprenorphine/naloxone). ◦ Coordination with Primary Care units. ◦ Implementation of new programs adapted to the circumstances of the alarm state. ◦ Information from professionals on the impact on patients of the alarm state: relapses, compliance with the pharmacological treatments, psychopathology's evolution if there is, beginning or increase in the alcohol consumption, benzodiazepines or other substances, changes in the “market” of drugs in their city. ◦ Patients and Professionals Affected by Covid-19 Infection. ◦ Degree of satisfaction expressed by users with the attention received. • In the Residential Facilities/Nursing homes: ◦ The continuity or not of the center's functioning. ◦ Changes in the operating rules. ◦ Execution or not of new admissions. ◦ Registration of the discharges: scheduled, voluntary, forced. ◦ Patients and Professionals Affected by Covid-19 Infection. ◦ Degree of satisfaction expressed by users with the attention received.

## Results

In all ODC/OAC/dual disorder programs and day centers, telework was applied, according to the contingency plan, patients could only be contacted by telephone or telematically. Only in the most urgent clinical cases, the on-site assistance was provided, taking extreme precautions and hygienic and protective measures. Most of the patients agreed on the telephone follow-up. There were no urine controls, with only some specific exceptions. Daily methadone release was discontinued in all centers except one unit, and only for a few not well-controlled patients. The patients only went to these units to collect methadone; they were given doses to cover 1, 2, or 3 weeks (even up to 4 weeks in one of the centers). Furthermore, in a specific area in this community (El Bierzo) a system was organized to bring the methadone dispensing closer to the patients. Seventeen new methadone treatments and 1 buprenorphine/naloxone treatment started in 6 different ODC.

Related to the pharmacological treatments, 3 centers were found administering the monthly injectable treatment to their patients (these patients had already been doing it regularly). The prescriptions for psychopharmacological and buprenorphine/naloxone treatments, were given out thanks to the good coordination of all units with the Primary Care system.

The Detoxification and Dual Disorder Inpatient Unit (DDDIU), located in Salamanca, which is a designated in Castile and Leon, was closed in the beginning of the confinement, in order to give up space to the COVID Rooms for the University Healthcare Complex of Salamanca (7). Likewise, the dispensing of methadone in Zamora's Healthcare Complex was suspended for the same reason. This activity was undertaken by the ODC of this province. Moreover, in Salamanca a program was accomplished to deal with mental health problems, including addictions, for homeless patients confined in a municipal center.

The professionals of 2 Therapeutic Communities (TC) and the 2 Alcoholic Rehabilitation Centers (ARC) of Castile and Leon who were interviewed, continue working with patients who were already admitted.

However, there were no new admissions, except for two patients, one from the ID-DDU in Salamanca and the other from the Psychiatric Service of the Río Hortega Hospital in Valladolid city. The patients who were on therapeutic leave at the time the state of alarm was declared were unable to return to the community. They kept in touch with them and their families over the phone, they will be offered readmission for follow up and treatment when the health authorities and the new contingency plan allows it. There were very few scheduled discharges (7), even some patients preferred to postpone their discharge. No voluntary or forced discharges were performed.

All residential centers had to ease or modify rules to adapt to the circumstances, for example, facilitate the calls to the families, etc. On the other hand family visits and outings were suspended. In all the units, the indications of the contingency plan of the Regional Commissioner for drugs in Castile and Leon have been followed (Table 4).

**Table 4:** Action proposals in DAACYL.

**Situation/measures**	**General measures**		**Locale cases**		**Cases among DAACYL professionals and/or users**	
	**Outpatient**	**Inpatient**	**Outpatient**	**Inpatient**	**Outpatient**	**Inpatient**
Social distancing and/or mask	✓	✓	✓	✓	✓	✓
Information line	✓	✓	✓	✓	✓	✓
Hand washing Coughing into the elbow Ventilation and cleaning	✓	✓	✓	✓	✓	✓
Do not attend to the center and contact the healthcare system if symptoms develop	✓	✓	✓	✓	✓	✓
Distance the time of collection of methadone	✓	✗	✓	✗	✓	✗
Replace face-to-face attention with telephone follow-up	✓	✗	✓	✗	✓	✗
Discontinuation of family visits	✓	✓	✓	✓	✓	✓
Temporary suspension of hospital admissions (minimum 14 days)	✗	✓	✗	✓	✗	✓

*DAACYL: Drug Addiction Assistance Network of Castile and Leon. Outpatient: centers for outpatient drug clinic; Outpatient alcohol clinic, Day centers. Inpatient: Therapeutic communities, alcoholic Rehabilitation Centers. Based on the contingency plan of the Regional Commissioner for drugs (March 2020)*.

According to the opinion expressed by the professionals surveyed, it was observed that the clinical impact in the first 6 weeks is moderate, which implies that not as many relapses and dropouts were detected as expected compared to the weeks with normal operation without a pandemic. The patients were stable, taking the medication appropriately without presenting clinical complications, even those with dual disorders.

Relapses also seem to be under control. Six centers detected that some patients increased or started consuming alcohol and benzodiazepines (especially alprazolam). In Salamanca's Outpatient Alcohol Clinic (OAC) relapses are detected on the basis of a clinical interview and, if possible, with urine controls in a protocolized manner every week. In this unit, at least 2 relapses and 3 exacerbations in alcohol consumption were detected, one of them required an urgent hospital admission due to acute organic distress; compared to 9 relapses that occurred after attending 144 patients in the week 15–21 April 2019. In the Outpatient Dual Disorder Program of Salamanca, 2 relapses were detected during the confinement period, during the second and fifth weeks.

In general patients describe that the consumption of illegal drugs has decreased, although some of them admit they still continue consuming. Several cases reported the price of cannabis increased these days.

Until May 11, 2020, the date on which the collection of information in the DAACYL centers and services ended, the impact of COVID-19 had been very low. In the case of users, 35 confirmed cases were declared, 72 probable cases pending confirmation and 4 deaths. It is significant that in the residential centers there were only 2 probable cases that were awaiting confirmation at the time of completing the information collection and no deaths. With regard to professionals, the impact was also very low: 6 confirmed cases, 10 probable cases and no deaths. The data referring to the volume of patients treated up to that moment in the centers and services were not collected, so it is not strictly possible to calculate the prevalence in patients. Regarding the professionals who provide service in the DAACYL, the prevalence of probable and confirmed cases was 4.78%.

## Discussion

The readjustment of the network has been very fast and consistent with the preliminary descriptions of the literature, such as reducing the face-to-face and hospital activity (5), deploying resources with telephone and online supports. The adaptation and use of telemedicine that has been implemented so suddenly in patients with addiction, seems to be working well. This adjustment has already been suggested by authors who have studied the pandemic in China (6, 8). There are previous international experiences, especially in the United States, on the use of telemedicine in patients with addictions (27, 28).

Possibly the distance and the type of health resources have facilitated its development. In Europe the experience is preliminary (29) and in Spain this experience is not developed in a massive scale. It has only been used in experimental programs and mostly with tobacco addiction (30).

The access to treatments with opiate agonists was simplified and was made more flexible, increasing the “take-home” system, doubling or quadrupling the number of days allowed, following the Castile and León contingency plan, with Spanish (20) and international recommendations, in America (21, 23), and Asia (22). Other suggested options, such as door-to-door delivery (21) were not implemented, although in large and uninhabited areas such as El Bierzo area, methadone dispensing was brought closer to further areas. Very few treatments with opioid agonists were introduced, due to difficulties in starting it, since the appointments could not be done regularly. In the future, the pharmacological approach and the interactions between psychotropic drugs and the drugs used in the treatment of COVID-19 patients should be considered (31). The interactions of antivirals and psychotropic drugs is known (12), but the complex combinations used for the treatment of COVID-19 is not known. The side effects during and after the treatment of COVID-19 is unknown, so they must be especially considered (8).

The DDDIU, located in Salamanca, which is the designated unit in Castile and Leon, was closed. There are no descriptions of this type of units in the literature. In the Chinese psychiatric units, the hospitalizations reported are shorter, with a stricter criteria for admission, the outpatient follow-up was the basis, they implanted isolation and visits were avoided (6).

Some of the measures, such as having minimum contact with the family on detoxification admissions, are already common in this units. However, the other measures are not very applicable. It should be noted that there are differences due that many of these units seem to be based in psychiatric hospitals (6). Some of the measures cited, such as the isolation of patients from the outside and the increase of telephone contacts, have been applied in residential centers.

The telephone interviews did not find the reported consequences of COVID-19 such as anxiety, depression, insomnia, suicide risk, poor adherence to treatment (6). Not even the specific risks in patients with addiction, including relapses, emergency department problems, and COVID-19 infections (10). Although these findings must be verified when the situation returns to normal. In the area where the homeless program was implemented, no emergency department visits and clinical decompensation was detected, but these programs have not been generalized around the territory.

Against the expected the frequent relapses and clinical decompensation that drug dependent people and dual patients present in normal conditions (32), are minimal nowadays, although drug consumption is still ongoing; since it is easier to acquire, the consumption of alcohol and anxiolytic drugs is possibly increasing.

The limited number of COVID-19 cases detected is surprising. These patients seem to be more vulnerable due to the organic and mental disorders associated, together with their lifestyle with low hygiene and self-control, the social exclusion they suffer, and their smoking addiction. It is possible that the infection is being underestimated, and that some patients have had the infection asymptomatically or with mild symptoms, since this population is accustomed to have withdrawal (33) or intoxication symptoms (34). Therefore, it is possible that the symptoms of COVID-19 have gone unnoticed. We contemplate that these patients have an immune system accustomed to different pathogens. We also discuss that some of the psychotropic drugs that these patients frequently take, such as methadone (35), other opiates (36), antipsychotics (37) or mood stabilizers (lamotrigine) (38) may have an anti-inflammatory effect. This could modulate the inflammatory effects produced by COVID-19, being one of the research lines in the treatment of this infection (39, 40).

The hypothesis that patients with addiction, who have experienced serious infections such as HIV, tuberculosis and viral hepatitis (41–43), have considered the severity and risks of this infection before the general population, adopting protective measures.

COVID-19 infection among DAACYL professionals is not very high, even being a population at risk. The high risk of acquiring the infection has been described in mental health professionals (2, 5, 7). The practice of telemedicine and not resorting to the units can explain this situation.

The response described is the initial one and it will change, however, it is relevant to plan long-term care incorporating the needs of everyone, professionals and different types of patients (11). Decisions must also be made to allow the continued attention and access to treatment, despite the current pandemic of COVID-19, or possible future ones. On the other hand, specific programs should be developed to prevent transmission among drug users, especially, through intravenous dissemination. Also avoiding the share of equipment for smoking, inhaling, vaping or injected drugs (13).

In the limitations of this study, the successful use of telemedicine in this situation could not possibly be the same as the normal attention, so the results should be viewed with caution. Probably, the presence of the COVID-19 infection in patients is being underestimated, this work is only a 6-week report. It was not possible to contact the smoking cessation programs, currently paused, since it is closely related to the Pneumology Service. However, this work is a real-world experience and can be useful to explain the complete response of a drug-addiction healthcare network. It would be important to consider it in the places where the infection is developing or for future measures, if the pandemic were to happen again.

We conclude that the response was assembled in a short time and the execution has been successful. At the moment the clinical response and the care system for people with substance use disorder have managed to control the situation in the drug units of Castile and León.

The use of telemedicine techniques in a pandemic situation for patients with addiction is encouraging. Its implementation *in situations* outside the crisis in Spain should be studied. However, these findings must be re-evaluated, since in the medium term the system cannot be paralyzed.

Further research should be carried out to study the reaction of the health system and the impact of COVID-19 on the course, treatment, prevalence and new approaches for patients with addictions. It is meaningful to prevent the development of behavioral addictions, the increase in the consumption of alcohol and benzodiazepines and the use of homemade preparations in confined patients suffering from addiction (14).

It is essential to move toward a progressive normalization in socio-sanitary assistance to drug dependent people. Always taking into account the recommendations of the health authorities to prevent the spread of the infection, while the impact on the health system begins to subside.

## Data Availability Statement

The raw data supporting the conclusions of this article will be made available by the authors, without undue reservation.

## Ethics Statement

Ethical review and approval was not required for the study on human participants in accordance with the local legislation and institutional requirements. Written informed consent for participation was not required for this study in accordance with the national legislation and the institutional requirements.

## Author Contributions

CR has been the main author of the article, designing, and elaborating the complete investigation process. AÁ-N, MG, LA, and BV-H have collected the clinical data necessary for its construction and have written fragments of it. MF-M and FM-G have revised the article bringing new perspectives. NC-E, SG-L, CL-Á, and RM have revised and improved the translation, design, and elaboration of the tables. All authors have contributed ideas and clinical experience for the discussion of the main topic.

## Conflict of Interest

CR declares that in the last years he has received remuneration for participating as a speaker in activities of Janssen-Cilag, Indivior, Lundbeck, Otsuka, Servier, GSK, Astra, Gilead, MSD, Sanofi, Exceltis, Abbvie, Takeda Rubio and Casein. He has received remuneration for participating as a consultant in meetings of Gilead, MSD, Mundipharm, INDIVIOR, Exceltis, Martindale, Camurus, Gebro and Abbive. It received funding for the Proteus project and the COSTEDOPIA project by Indivior. He has received Gilead medical education scholarships. BV-H declares that in the last years she has received remuneration as a speaker for Janssen-Cilag and Lundbeck. NC-E declares that in the last years she has received an economic prize from the Janssen-Cilag oral communications competition and received remuneration as a speaker for Sanofi. MF-M declares that in the last years she has received remuneration as a speaker for Sanofi. MF declares that he has carried out applied research studies (clinical trials mainly) and speaker at activities carried out by the following companies in recent years: Janssen-Cilag, MSD, Lundbeck, Otsuka, Servier, Acadia, Pfizer, Roche. The remaining authors declare that the research was conducted in the absence of any commercial or financial relationships that could be construed as a potential conflict of interest.
